# Nongenomic mechanisms of physiological estrogen-mediated dopamine efflux

**DOI:** 10.1186/1471-2202-10-59

**Published:** 2009-06-16

**Authors:** Rebecca A Alyea, Cheryl S Watson

**Affiliations:** 1 Department of Biochemistry & Molecular Biology, University of Texas Medical Branch, Galveston, TX 77555-0645, USA

## Abstract

**Background:**

Neurological diseases and neuropsychiatric disorders that vary depending on female life stages suggest that sex hormones may influence the function of neurotransmitter regulatory machinery such as the dopamine transporter (DAT).

**Results:**

In this study we tested the rapid nongenomic effects of several physiological estrogens [estradiol (E_2_), estrone (E_1_), and estriol (E_3_)] on dopamine efflux via the DAT in a non-transfected, NGF-differentiated, rat pheochromocytoma (PC12) cell model that expresses membrane estrogen receptors (ERs) α, β, and GPR30. We examined kinase, ionic, and physical interaction mechanisms involved in estrogenic regulation of the DAT function. E_2_-mediated dopamine efflux is DAT-specific and not dependent on extracellular Ca^2+^-mediated exocytotic release from vesicular monoamine transporter vesicles (VMATs). Using kinase inhibitors we also showed that E_2_-mediated dopamine efflux is dependent on protein kinase C and MEK activation, but not on PI3K or protein kinase A. In plasma membrane there are ligand-independent associations of ERα and ERβ (but not GPR30) with DAT. Conditions which cause efflux (a 9 min 10^-9 ^M E_2 _treatment) cause trafficking of ERα (stimulatory) to the plasma membrane and trafficking of ERβ (inhibitory) away from the plasma membrane. In contrast, E_1 _and E_3 _can inhibit efflux with a nonmonotonic dose pattern, and cause DAT to leave the plasma membrane.

**Conclusion:**

Such mechanisms explain how gender biases in some DAT-dependent diseases can occur.

## Background

### DAT diseases, function, and connection to hormonal states

Parkinson's, Tourette's, attention deficit hyperactivity disorder (ADHD), Alzheimer's, and schizophrenia are all associated with alterations in dopamine-driven function involving the dopamine transporter (DAT) [1]. The DAT belongs to a family of Na^+^/Cl^- ^dependent plasma membrane symporters whose function is to rapidly remove dopamine from the synaptic space, resulting in the termination of neurotransmitter signaling. Alterations in the location and function of the DAT can lead to changes in dopamine signaling affecting behavioral outcomes and also increased susceptibility to neuronal insult [2]. Females are more susceptible to the onset or exacerbations of these diseases during life stages when female hormonal fluctuations and changes are most pronounced (adolescence, premenopausal female cycling, perimenopause, and postmenopause), which suggests that changes in physiological estrogen levels can influence neurochemical pathways including dopamine signaling [3-6]. Many studies have linked 17β-estradiol (E_2_), the predominant physiological estrogen, to neuroprotective properties, but the mechanisms of action on the DAT system are not fully elucidated, and may differ depending upon the levels of E_2 _administered and the actions of other estrogens.

### Nongenomic effects of E_2 _on the DAT

Recent attention to the nongenomic actions of E_2 _can provide some additional insight as to its effect on the DAT system. E_2 _is produced by the ovaries and reaches all tissues by the circulation, but in the brain it is also produced by conversion of androgens via the enzyme aromatase which is enriched in mammalian presynaptic boutons [7]. This creates an environment for increased rapid bioavailability of E_2 _which can elicit nongenomic effects such as Ca^2+ ^mobilization, kinase activation, and alterations in dopamine subcellular location via membrane estrogen receptors (mERs) [8-11]. We have previously examined a well characterized non-transfected neuronal cell culture model (PC12 cells) that expresses three known mERs: mERα, mERβ, and GPR30; in these cells physiological levels of E_2 _[8,12] and low levels of xenoestrogens [13] can rapidly reverse actions of the DAT.

Modifications in the phosphorylation state of the DAT by kinases causes alterations in the function and location of the DAT [reviewed in [14]]. Amphetamine, a psychostimulant, also causes reversal and altered cellular location of the DAT which is known to be regulated by kinases, phosphatases, and Ca^2+ ^localization and association [15]. Therefore, we hypothesized that the estrogen-mediated changes in dopamine efflux that we have observed [8,12] may involve similar mechanisms. In this study we examined both indirect and direct mechanisms involved in physiological estrogen-mediated dopamine efflux in conjunction with the cellular location of the ERs and the DAT. We studied the involvement of protein kinases A and C (PKA, PKC), phospho-inositol 3 kinase (PI3K), extracellular-regulated kinases (ERKs), vesicular release of dopamine, and changes in intracellular Ca^2+ ^concentrations in the actions of estrogens. Then we addressed the subcellular localization of ERα, ERβ, the alternative membrane ER (GPR30), and DAT to see if estrogen-induced trafficking of these proteins in and out of the plasma membrane could explain some of the regulatory effects on dopamine efflux. In addition to E_2_, we also examined the effects of estrone (E_1_, at high levels postmenopausally) and estriol (E_3_, at high levels during pregnancy) to see if these estrogens may have some potent nongenomic signaling effects of their own, as we have previously observed in pituitary cells [16], and if they can also affect DAT function. These differential regulatory effects on DAT by different physiological estrogens may provide some insights into mechanisms controlling the incidence of neurological diseases during life stages accompanied by fluctuations or change in the steady state levels of these hormones.

## Methods

### PC12 cell culture

PC12 cells were grown in high-glucose, phenol red-free RPMI 1640 medium containing 5% fetal bovine serum (FBS) and 5% equine serum (HS). To promote PC12 differentiation and minimize the effects of endogenous hormones respectively, 20 ng/ml NGF-β was added in medium supplemented with 0.5% of 4× charcoal-stripped FBS and HS for 48 hrs prior to experiments.

### Dopamine efflux assay

We measured ^3^H-dopamine efflux using selective catecholamine transporter inhibitors to define specific dopamine transport via the DAT as previously described in [8]. PC12 cells were plated on poly-D-lysine (10 μg/ml)-coated 48-well plates and uptake buffer (25 mM HEPES, 120 mM NaCl, 5 mM KCl, 2.5 mM CaCl_2_, 1.2 mM MgSO_4_, 1 μM pargyline, 2 mg/ml glucose) containing 0.2 mg/ml ascorbic acid, and desipramine (50 nM), pH 7.4 ± GBR 12909 was added for 60 min at 37°C. In experiments containing 50 nM reserpine, a VMAT inhibitor, a 120 min preincubation in uptake buffer preceded the 60 min GBR 12909 preincubation. GBR 12909 (100 nM) was added to define selective efflux by DAT. In experiments containing kinase inhibitors 10 μM U0126 (a MAPK inhibitor) or 10 μM Ly294002 (a PI3K inhibitor) were also added during the 60 min uptake buffer addition. 10 μM H89 (a PKA inhibitor) and 100 nM Ro32-0432 (a PKC inhibitor) were added to the uptake buffer for 30 min of preincubation. For experiments testing Ca^2+^involvement, 1 μM thapsigargin was added for a 15 min preincubation to empty intracellular Ca^2+ ^stores, or cells were incubated for 10 min in 0 Ca^2+ ^medium (5 mM EGTA, 10 mM HEPES, 130 mM NaCl, 5.5 mM KCl, 2 mM MgCl_2_, 7 mM glucose, 30 mM sucrose, pH 7.4) and washed twice in 0 Ca^2+ ^medium. For all assays cells were loaded with ^3^H-DA (20 nM) for 10 min prior to two washes in release buffer (25 mM HEPES, 120 mM NaCl, 5 mM KCl, 1.2 mM MgSO_4_, 1 μM pargyline, 2 mg/ml glucose, 0.2 mg/ml ascorbic acid, and 50 nM desipramine). Release buffer containing treatments, +/- GBR12909, was then added, and extracellular fluid was collected at 9 min to assess^3^H-DA efflux. Triplicate aliquots were counted in 2 ml Scintiverse II scintillant using a Beckman LS600SE scintillation counter. Specific efflux was defined by averaging the disintegrations per minute due to efflux in the presence of desipramine and GBR 12909, and then subtracting these values from the efflux observed with desipramine alone. We subtracted background (vehicle controls) from treatment groups and represented the data as ^3^H-DA efflux compared to % of 9 min 10^-9 ^M E_2_-induced efflux (set as 100%).

### Co-Immunoprecipitation

PC12 cells were collected from five, 150 cm^2 ^Corning tissue culture flasks by scraping, and then centrifuged at 1500 × g, 4°C for 5 min, and resuspended in 2 ml homogenizing buffer (10 mM Tris-HCl, 1 mM EDTA, 1 mM EGTA, pH 7.4). Cells were then sonicated 15 times using a pulse probe sonicator, and further processed using a Dounce homogenizer, on ice, until the majority of cells appeared broken by microscopic examination. The resulting broken cell preparation was then centrifuged at 1500 × g at 4°C to remove the nuclear pellet. The supernatant was then centrifuged at 120,000 × g at 4°C to obtain the plasma membrane pellet, which was then resuspended in membrane buffer (50 mM Tris-HCl, 1 M NaCl, 5 mM KCl, 2 mM CaCl_2_, 1 mM MgCl_2_, 250 mM sucrose, 1 mM EDTA, 1 mM DTT, 1:1000 protease inhibitor cocktail, pH 7.4) by stirring 8 hours at 4°C and then re-pelleted by centrifugation for 45 min at 45,000 × g, 4°C. The Bradford Bio-rad assay was used to determine protein concentration in the supernatant per manufacturer's instructions. Protein samples were incubated with 40 μl protein G agarose (Sigma, P-7700) beads for 10 min at 4°C, then centrifuged using a microfuge for 1 min. The supernatant was incubated overnight at 4°C with 2.5 μg DAT antibody (C-20, Santa Cruz: sc-1433). 50 μl of protein G agarose beads were washed 3 times in phosphate buffered saline (PBS) and samples containing antibody were incubated with these beads for 4 hours at 4°C on a rotator. Beads were then washed 4 times with PBS for 10 min, each wash. Samples were eluted using 50 mM glycine buffer pH 2.5, added to SDS sample buffer and heated at 67°C for 10 min, and then electrophoresed on a 7.5% acrylamide SDS-PAGE gel followed by transfer to a nitrocellulose membrane. Blots were blocked using 2.5% BSA and 2.5% milk in 10 mM Tris-buffered saline, pH 7.4, for 1 hr before overnight incubation with primary antibodies (Abs), to ERα (1:1000 Mc-20, Santa Cruz: sc-542), ERβ (1:2000 Clone 9.88, Sigma: E1276), GPR30 (1:1000 Novus: NLS4271), and DAT (1:1000, Santa Cruz: sc-33056) at 4°C. Blots were washed three times for 15 mins with 0.05% TBST and incubated for 1 hr with peroxidase-conjugated anti-mouse IgG (Amersham Biosciences) for ERα and ERβ, or peroxidase-conjugated anti-rabbit IgG (Amersham Biosciences) for GPR30, or peroxidase-conjugated anti-goat (Amersham Biosciences) for DAT. Immunoreactivity was detected by enhanced chemiluminescence (Amersham Biosciences) on Hyperfilm (Amersham) film.

### Quantitative plate immuno-assay

Briefly, PC12 cells were plated on poly-D-lysine (10 μg/ml)-coated 96-well plates at 5000 cells per well, as previously described [8]. NGF-differentiated, serum-deprived cells were washed with PBS for 5 min, and treatments were added in the above uptake buffer with 50 nM dopamine for 9 min. Cells were fixed for 30 min at room temperature with 50 μl 2% paraformaldehyde, and 0.2% gluteraldehyde +/- NP-40 to permeabilize or not permeabilize cells, respectively. Cells were then washed twice (5 min each) with PBS and blocked with 0.1% fish gelatin/PBS for 45 mins at 22°C. Diluted 1° Abs, to ERα (1:1000 Mc-20, Santa Cruz: sc-542), ERβ (1:1500 Clone 9.88, Sigma: E1276), GPR30 (1:1000 Novus: NLS4271), and DAT (1:2000 W-17, Sigma: sc-33056) were added overnight at 4°C; 2 μg anti-clathrin Ab provided a control for cell permeabilization [8]. Cells were washed three times in PBS and incubated in appropriate biotinylated 2° Ab for 1 hr, then washed three times prior to 60 min incubation with ABC-alkaline phosphatase (AP) solution. Cells were washed five times with PBS, and the substrate para-nitro-phenol phosphate (pNpp) plus 0.5 mM levamisole was added in 100 mM sodium bicarbonate solution for 30 mins at 37°C. Plates were read at A_405 _nm and then rinsed and stained with 0.1% crystal violet for 30 mins at room temperature, then washed with ddH_2_0 and dried overnight. Dye was then extracted from each well with 50 μl 10% acetic acid, read at A_590_, and used to estimate cell number per well. Data are plotted as % of vehicle-treated control levels (A_405 _nm/A_590 _nm).

### Statistics

Statistical analyses for all assays were performed using SigmaStat software (Chicago, IL, USA), and statistical significance was accepted at p < 0.05. Figure legends contain the n for each experimental set and the specific statistical analysis applied. All experiments were repeated 3 times.

## Results

### PKC and MAPK are involved in E_2_-mediated dopamine efflux

**Figure 1:**
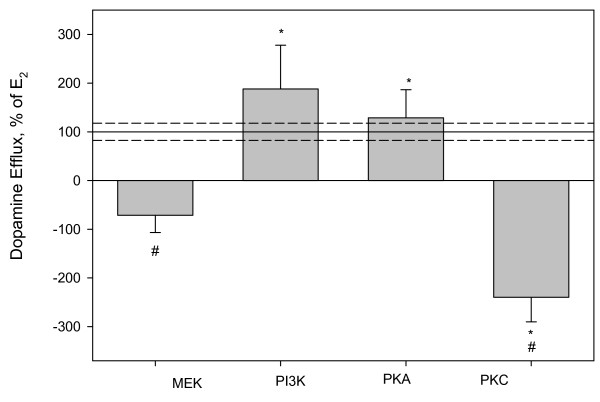
**^3^H-DA efflux assay after a 9 min 10^-9 ^M E_2 _treatment in the presence of kinase inhibitors**. A) U0126 is a MEK inhibitor, LY294002 is a PI3K inhibitor, H89 is a PKA inhibitor, and Ro 32-0432 is a PKC inhibitor. The Y-axis is % of 10^-9 ^M E_2_dopamine efflux response at 9 mins, dashed lines are errors around the mean. * = p < 0.05 significance compared to control, # = p < 0.05 from E_2 _treatment, n = 24 in 3 experiments.

We have previously demonstrated that a 9 min 10^-9 ^M E_2 _treatment causes DAT specific dopamine efflux in non-transfected NGF-differentiated PC12 cells expressing ERα, ERβ, and GPR30 [8]. This led us to use this model to first explore the possible control of E_2_-mediated dopamine efflux by the most often reported mechanism, kinase involvement. Many kinases including PI3K, PKA, mitogen-activated protein kinases (MAPKs), and PKC are known to regulate DAT activity, specifically amphetamine-induced dopamine efflux, and DAT location [17-20]. We pre-incubated PC12 cells with inhibitors for PKC, MAPK/ERK kinases (MEK), PKA, or PI3K, using optimal preincubation times for each inhibitor (see materials and methods), and then added 10^-9 ^M E_2 _for 9 mins prior to measuring dopamine efflux. Figure 1 shows that inhibiting either MEK or PKC significantly inhibited E_2_-mediated dopamine efflux. Inhibiting PI3K or PKA did not affect E_2_-mediated dopamine efflux.

### The presence of intracellular Ca^2+ ^is required for E_2_-mediated dopamine efflux

**Figure 2:**
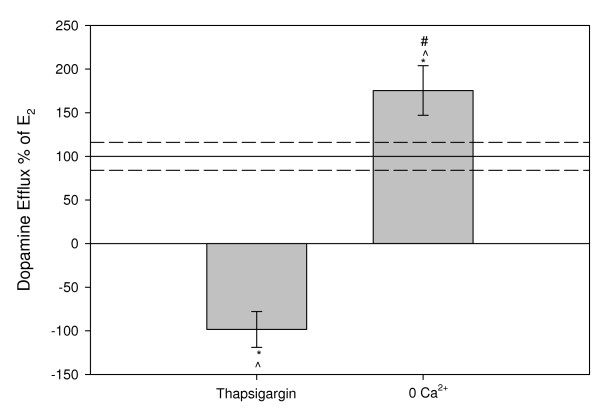
**^3^H-DA efflux assay after a 9 min 10^-9 ^M E_2 _treatment in the presence of Ca^2+ ^depleted medium compared to normal efflux medium**. A 15 minute pretreatment with thapsigargin releases intracellular Ca^2+ ^stores; 0 Ca^2+ ^media removes extracellular Ca^2+ ^from the treatment. The Y-axis is % of 10^-9 ^M E_2 _dopamine efflux response at 9 mins, dashed lines are errors around the mean. * = p < 0.05 significance compared to control, # = p < 0.05 vs. thapsigargin, ^ = p < 0.05 vs. normal efflux medium, n = 24 in 3 experiments.

Although we have controlled for dopamine flux specifically through the DAT through the use of DAT- and norepinephrine-selective transporter inhibitors, the addition of these inhibitors does not account for the possibility of exocytotic release of dopamine which is dependent on extracellular Ca^2+^. Intracellular Ca^2+ ^is also an important second messenger signal that is required to activate Ca^2+^-dependent PKC isoforms. Compared to 9 min 10^-9 ^M E_2 _treatment (Figure 2), preincubating the cells for 10 min in 0 Ca^2+ ^medium containing 5 mM EGTA did not inhibit E_2_-induced dopamine efflux, but instead actually increased dopamine efflux. However, the prior emptying of intracellular stores of Ca^2+ ^with thapsigargin did reverse E_2_-mediated dopamine efflux.

### Vesicular release of dopamine is not involved in E_2_-mediated dopamine efflux

**Figure 3:**
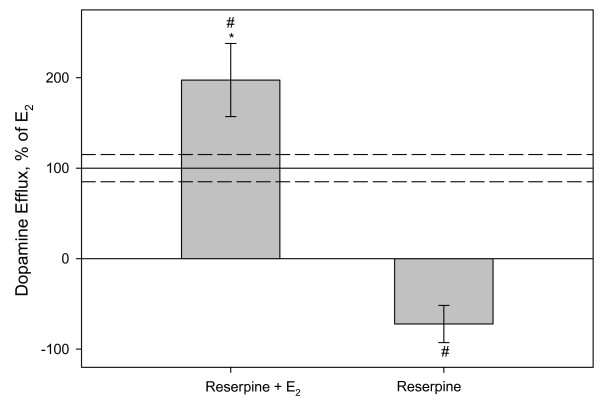
**^3^H-DA efflux assay after 9 min 10^-9 ^M E_2 _treatment in the presence of VMAT inhibitor**. PC 12 cells were pretreated for 2 hrs with reserpine, a VMAT inhibitor, in order to inhibit vesicular release of DA, and then treated with 10^-9 ^M E_2 _or ethanol (vehicle control). The Y-axis is % of 10^-9 ^M E_2 _dopamine efflux response at 9 mins, dashed lines are errors around the mean. * = p < 0.05 significance compared to control, # = p < 0.05 vs. E_2 _treatment without reserpine. n = 18 in 3 experiments.

We then further examined the mechanisms involved in the E_2_-induced movement of dopamine to the outside of PC12 cells. To confirm that vesicular release of dopamine is not involved in E_2_-mediated dopamine efflux mechanism, we preincubated our cells with reserpine, a vesicular monoamine transporter (VMAT) inhibitor which causes emptying of dopamine from VMATs. Figure 3 shows that the inhibition of vesicular release does not inhibit subsequent E_2_-induced dopamine efflux (and that the inhibitor alone does not cause dopamine efflux), further confirming that the E_2_-mediated dopamine efflux that we have observed is specifically via the DAT. We found that the dopamine efflux resulting from treatment with reserpine alone compared to the control (in the absence of E_2_) are similar (within the same error around zero) indicating that basal and reserpine control are not different from one another. We also noted that inhibiting VMATs significantly increased E_2_-mediated dopamine efflux.

### Effects of three physiological estrogens on dopamine efflux and trafficking of the DAT and ERs

**Figure 4:**
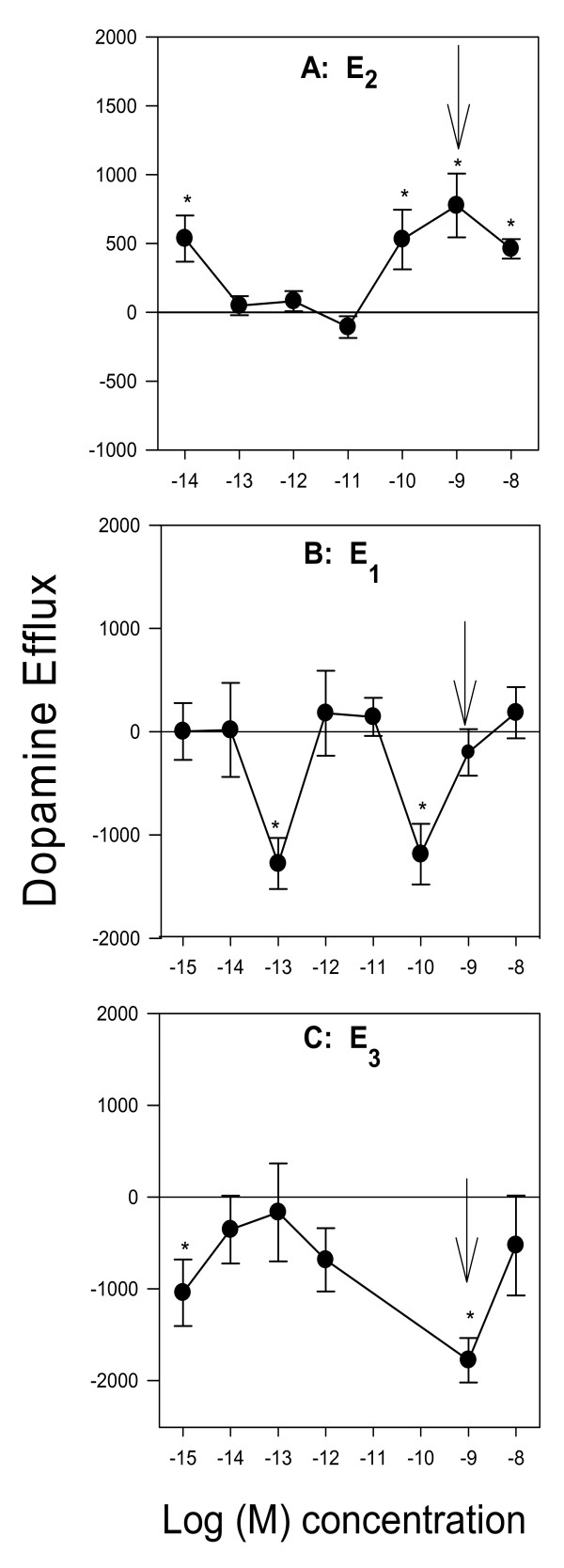
**Concentration-dependent dopamine efflux patterns for E_2_, E_1_, and E_3 _at 9 min (A) E_2 _(B) E_1 _(C) E_3_**. Arrows indicate the concentration chosen for quantitative plate assay (Fig 5) ERα, ERβ, and GRP30 levels after 9 min 10^-9 ^M E_2 _treatment were previously reported in [8] * = p < 0.05 from control, # = p < 0.05 from membrane, and are shown here for comparison to changes caused by E_1 _and E_3_. n = 24 in 3 experiments

**Figure 5:**
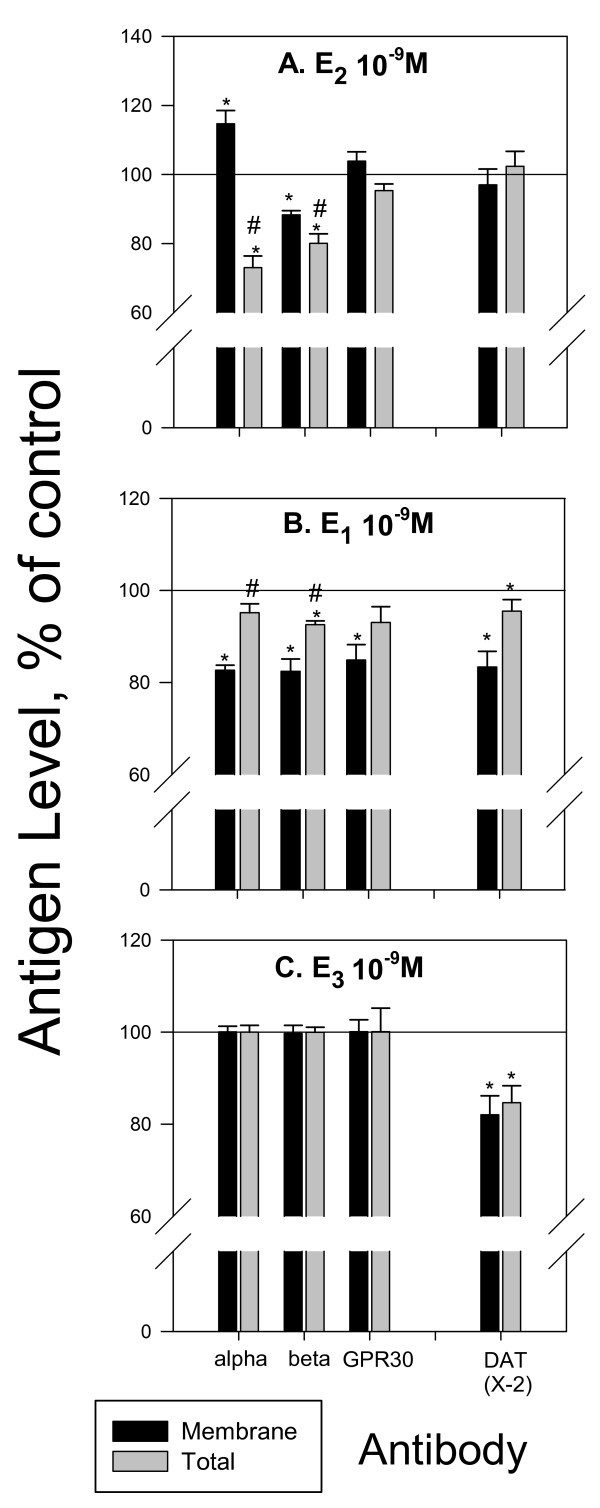
**Quantitative plate assay measuring immunoreactive protein levels for plasma membrane and total ERα, ERβ, GPR30, and DAT after 9 min E_2_, E_1_, and E_3 _treatment**. (A) E_2 _(B) E_1 _(C) E_3_. ERα, ERβ, and GRP30 levels after 9 min 10^-9 ^M E_2 _treatment were previously reported in [8] * = p < 0.05 from vehicle control, # = p < 0.05 from membrane, and are shown here for comparison to changes caused by E_1 _and E_3_. n = 24 in 3 experiments

Changes in DAT membrane presence and functioning could be an important mechanism for alterations in neurochemical signaling by several physiological estrogens (not just E_2_). Therefore, we first monitored the concentration-dependent effects of a 9 min physiological estrogen (E_2_, E_1_, or E_3_) treatment on dopamine efflux (Figure 4). E_2 _(Figure 4A), caused dopamine efflux at 10^-14 ^M followed by a return to baseline, and then another peak of dopamine efflux at the higher concentrations (100 pM-10 nM). E_1 _(Figure 4B) and E_3 _(Figure 4C), did not cause dopamine efflux at the tested concentrations at 9 min but at 10^-13 ^and 10^-10 ^M E_1 _significantly inhibited dopamine efflux. E_3 _(Figure 4C) also did not cause dopamine efflux, but did cause inhibition at 10^-15^, and 10^-9 ^M concentrations with no effect at other concentrations. These bimodal concentration effects of estrogens on dopamine efflux are typical of nongenomic actions that we have described before on these and other cell types [8,21,22]. Such changes in dopamine efflux could be due to effects of estrogens on the trafficking of the DAT, and mERs (that are likely to regulate the DAT) to or from the plasma membrane, which we then investigated, shown in Figure 5. We selected the 10^-9 ^M concentration of each estrogen treatment at 9 min to investigate these possible effects (shown by arrows in Fig 4) because this is a physiological level for each [23], and because they cause distinctively different effects on efflux by the different hormones. E_2 _at this concentration, which had caused increases in efflux, increased the amount of ERα and decreased the amount of ERβ in the plasma membrane (Figure 5A). DAT membrane levels were unchanged. E_1 _treatment caused trafficking of all three ERs and the DAT away from the plasma membrane (Figure 5B) perhaps removing them from their place of association and functional influence. E_3 _treatment which caused inhibition of efflux did cause removal of plasma membrane DAT, but trafficking of the ERs was not affected (Figure 5C).

### The DAT directly associates with ERα and ERβ in the plasma membrane

**Figure 6:**
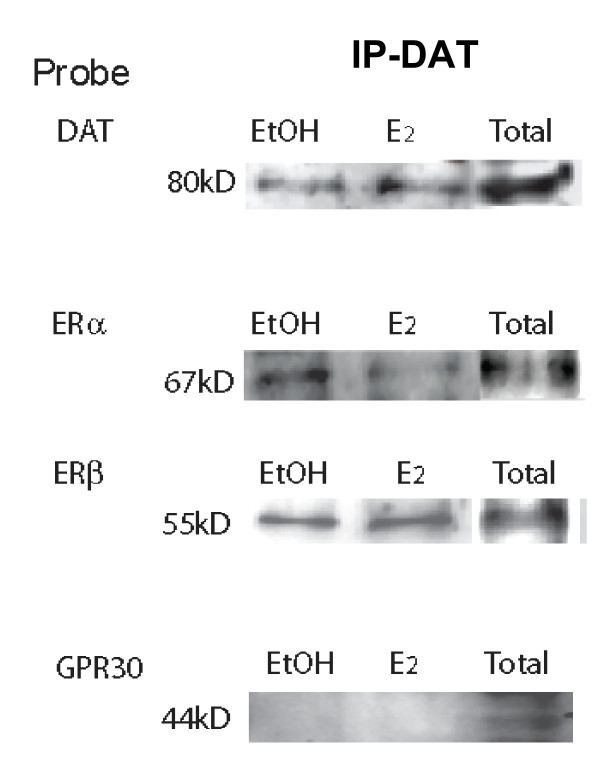
**Direct association of dopamine transporter and estrogen receptors on the plasma membrane vs. in total protein**. Co-immunoprecipitation of dopamine transporter after 9 min 10^-9 ^M E_2 _treatment and subsequent probing with antibodies for DAT, ERα, ERβ, and GPR30. This is a representative immunoblot of 3 experiments

We have previously reported that ERα is the predominant receptor mediator of E_2 _effects on dopamine efflux [8]. Therefore, we next tested for the direct interaction between the DAT and ER proteins in the plasma membrane at a time (9 mins) and concentration (10^-9 ^M) of optimal hormone-mediated dopamine efflux (Figure 6). In vehicle-treated (EtOH) control samples the pull-down pattern suggests a ligand-independent association of ERα and ERβ with the DAT. That is, plasma membrane-enriched fractions immunoprecipitated with a DAT antibody, co-immunoprecipitated ERα and ERβ, but not GPR30. We also tested for the presence of each ER and the DAT in plasma membrane total fractions and showed that each protein of interest was present (total). After E_2 _treatment ERα and ERβ are still present in the DAT pull-down, and GPR30 remains absent. A slight reduction in the amount of ERα is seen after E_2 _treatment. Therefore, prior to and immediately following E_2 _treatment, ERα and ERβ are associated with the DAT, which indicates a potential for a significant level of control between estrogens and the DAT.

## Discussion

Our studies pinpoint the contributions of regulatory kinase cascades and specific sources of regulatory Ca^2+ ^ions in the mechanisms of estrogenic control of the DAT. In addition, we demonstrate a role for other physiological estrogens besides E_2 _in regulating the function/subcellular localization of the DAT, and a physical association of two ERs (α and β) with the DAT before and during estrogen action. Such findings lay the basis for understanding how estrogen profiles associated with different life stages of women may influence processes and diseases associated with DAT function.

Previous *in vivo *studies have reported conflicting results on the hormonal regulation of DAT expression. One finding reports that E_2 _up-regulates DAT while others have shown that E_2 _down-regulates DAT expression [24,25]. Although, alteration in DAT expression leads to modifications in the capacity for a neuron to transport dopamine causing a decrease or increase in neurotransmitter signaling, we are reporting for the first time the nongenomic and acute mechanisms by which estrogens can regulate the DAT function.

Our data indicate that E_2_-mediated dopamine efflux is carrier-mediated transport based on our finding that it is dependent upon endogenous Ca^2+^, and that inhibition of exocytotic release does not inhibit hormone stimulated dopamine efflux. When inhibiting VMAT storage vesicles we observed an increase in E_2_-mediated dopamine efflux. Exocytotic release of dopamine via VMAT trafficking is dependent upon exogenous Ca^2+^, but reserpine, a VMAT inhibitor, causes emptying of dopamine from VMATs leading to increased levels of intracellular dopamine. We hypothesize that our observed level of increased efflux could be due to an increase in the concentration gradient of intracellular dopamine, thus facilitating dopamine efflux. Previous studies have shown that Ca^2+^-free medium does not alter baseline DAT uptake properties [26], further supporting our conclusion that this estrogenic effect is on transporter-mediated dopamine efflux. However, the removal of extracellular Ca^2+ ^caused a significant increase in E_2_-induced dopamine efflux which suggests extracellular Ca^2+ ^sensitive kinase activation or phosphatase activity might play a role in regulating E_2_-mediated dopamine efflux. Calcium/calmodulin dependent kinase II (CaMKII) activity and association with the DAT is known to be important for syntaxin 1A association with DAT and AMPH-mediated dopamine efflux [27]. Syntaxin 1A can regulate ion channels and neurotransmitter transporters [28], so the removal of extracellular Ca^2+ ^could disrupt CaMKII and syntaxin 1A association and thus affect estrogen-mediated efflux at this level. Future studies will further explore the mechanistic relationship between E_2_-mediated dopamine efflux and CaMKII and how this mechanism may resemble AMPH-mediated dopamine efflux.

Using inhibitors for a series of kinases, we found that both PKC and MEK are important for E_2_-mediated dopamine efflux. The DAT contains many PKC consensus sites and PKC activity is also important for the interaction of many of the DAT-associated proteins that control its location and activity. AMPH-mediated dopamine efflux is dependent primarily on a Ca^2+ ^sensitive PKC isoform, PKCβ [17]. Because E_2 _and AMPH both require intracellular Ca^2+ ^and PKC activity, it could be an interesting common point of regulation suggesting similar mechanisms of control. MEK and its downstream kinases are known to be one aspect of controlling trafficking of the DAT to and from the plasma membrane. In our experiments E_2 _did not change the subcellular location of the DAT, though the other tested estrogens did at the nM concentrations tested. Most likely our effects of E_2_-mediated dopamine efflux were mediated by a PKC-dependent mechanism. It is also possible that MEK cascade activation is secondary via dopamine signaling. D_2 _receptor activation by dopamine leads to MAPKs activation and increased intracellular Ca^2+^, which in turn also activates PKC [29]. We have previously reported that E_2 _also activates ERK in other cell systems [9,11].

We previously reported that E_2 _causes rapid dopamine efflux via mER activation, specifically by ERα liganding, with inhibitory regulation from ERβ and GPR30, accompanied by no change in plasma membrane levels of the DAT [8]. Regulation that removes DAT from the plasma membrane could alter both dopamine uptake and efflux, which in turn could lead to prolonged signaling changes due to altered synaptic dopamine levels. Other studies have shown that an increase in the presence of membrane DAT levels is an indicator of increased susceptibility to neurotoxins that are transported by the DAT; this creates an environment for increased uptake of synaptic dopamine which if not sequestered in VMATs, could increase intracellular reactive oxygen species (ROS) levels. E_1_, which is increased following menopause, does not cause dopamine efflux at the tested physiological concentrations in our studies, but does cause trafficking of the DAT and all three ERs (ERα, ERβ, and GPR30) from the plasma membrane. E_3_, a hormone which is high during pregnancy did not cause dopamine efflux, but at a physiological concentration significantly inhibited dopamine efflux while allowing retention of all three ERs at the plasma membrane. Since DAT plasma membrane levels controlling function determine the level of available synaptic dopamine, and E_1 _and E_3 _both cause removal of membrane DAT and inhibition of dopamine efflux, we speculate that this could account for some mood alterations during times of these hormonal fluctuations. E_3 _not only removes DAT from the membrane but reduces the total cellular DAT content. Because E_2 _and E_1 _treatment changed the subcellular location of the ERs to varying degrees, it is possible that these protein movements could alter or destabilize associations with the DAT which we will test in future studies.

We observed ligand-independent association of ERα and ERβ (but not GPR30) and DAT in vehicle-treated samples, while a 10^-9 ^M E_2 _treatment decreased (but did not eliminate) association between ERα and the DAT. Both the DAT and ERs are reported to be located within caveolin-containing lipid rafts in the plasma membrane, so these associations are not surprising. Our co-IP studies were designed to monitor if there is an association between the ERs and the DAT, but in order to determine if or how E_2 _treatment quantitatively caused changes in this association, further approaches are needed.

Conflicting studies have reported both increases and decreases in DAT levels in ADHD patients which indicate that other factors are involved. Stimulants that block DAT function are used in treatment regiments for ADHD resulting in improved inattention measurements [29]. During the follicular phase of the menstrual cycle females are more responsive to stimulants such as amphetamine, which suggests that the effects of estrogens and stimulants that target DAT interact [30-32].

## Conclusion

The significance of estrogen-coupled regulation of the DAT by both direct and indirect (kinase-mediated) interactions between ERs and the DAT should provide insights into how neurological diseases which involve the DAT are related to developmental, gender, and life stage issues. Now that we are beginning to mechanistically explore this system using well defined cell models, we will be able to ask more specific questions in *in vivo *systems relating to disease states. Such regulation may suggest new ideas about treatment and prevention of diseases associated with extreme hormonal fluctuations such as in postpartum depression.

## Abbreviations

Ab: antibody; DAT: dopamine transporter; E_2_: 17β-estradiol; E_3_: estriol; E_1_: estrone; ERα: estrogen receptor α; ERβ: estrogen receptor β; GBR 12909: 1-(2-[bis(4-Fluorophenyl)methoxy]ethyl)-4-(3-phenylpropyl)piperazine dihydrochloride; GPR30: G-protein coupled receptor 30; mER: membrane estrogen receptor; NGF: nerve growth factor; PC12 cells, pheochromocytoma cell line; ROS: reactive oxygen species; VMATS: vesicular monoamine transporters

## Authors' contributions

RAA and CSW contributed equally to the preparation of this manuscript and have approved the final versions.
